# The Impact of Noise and Brightness on Object Detection Methods

**DOI:** 10.3390/s24030821

**Published:** 2024-01-26

**Authors:** José A. Rodríguez-Rodríguez, Ezequiel López-Rubio, Juan A. Ángel-Ruiz, Miguel A. Molina-Cabello

**Affiliations:** 1 Department of Computer Languages and Computer Science, University of Málaga, 29071 Málaga, Spain; joseantoniorodriguez@uma.es (J.A.R.-R.); juanan7999@uma.es (J.A.Á.-R.); 2 Instituto de Investigación Biomédica de Málaga y Plataforma en Nanomedicina-IBIMA Plataforma BIONAND, 29009 Málaga, Spain

**Keywords:** deep learning, object detection, noise, brightness

## Abstract

The application of deep learning to image and video processing has become increasingly popular nowadays. Employing well-known pre-trained neural networks for detecting and classifying objects in images is beneficial in a wide range of application fields. However, diverse impediments may degrade the performance achieved by those neural networks. Particularly, Gaussian noise and brightness, among others, may be presented on images as sensor noise due to the limitations of image acquisition devices. In this work, we study the effect of the most representative noise types and brightness alterations on images in the performance of several state-of-the-art object detectors, such as YOLO or Faster-RCNN. Different experiments have been carried out and the results demonstrate how these adversities deteriorate their performance. Moreover, it is found that the size of objects to be detected is a factor that, together with noise and brightness factors, has a considerable impact on their performance.

## 1. Introduction

The constant presence of Gaussian noise, among other noises, in data processed by electronic devices is a phenomenon inherent to our highly technological society. This noise, closely linked to electromagnetic radiation, inevitably infiltrates any device that requires electrical communication, posing a constant challenge to the integrity of the data we process. In particular, image processing is significantly affected by this type of noise, since the vast majority of sensors used in this field are exposed to its influence. These sensors play a crucial role in a wide range of applications, especially in the life sciences. From dental imaging to digital mammography and ophthalmological examinations to assess eye health and analyze pathologies, electronic sensors are essential. However, the real problem arises when the images captured by these sensors are affected by Gaussian Noise, which can lead to alterations in the conclusions drawn from their processing [1].

This challenge is compounded in the field of Deep Learning, a discipline that has gained increasing prominence in the field of Artificial Intelligence. In this context, neural networks emerge as the fundamental pillars of this technological revolution. These networks appear as powerful tools that allow complex tasks to be tackled more efficiently in terms of time and resources, offering highly accurate results. Their application is diverse, ranging from event prediction to the simulation of complex systems, pattern recognition and classification, and system monitoring [2].

Among the rich variety of neural network models available, Convolutional Neural Networks (CNNs) stand out for their effectiveness in detecting objects in images and videos, analyzing medical images, processing natural language and creating recommendation systems, among other applications. These networks, specifically designed to process data in image format, are an essential tool in fields as diverse as medical diagnosis, autonomous driving and traffic management [3]. In particular, the object detection problem is a field where many applications are being developed [4,5].

However, the relationship between images processed by electronic sensors and neural networks brings with it a major challenge: the introduction of noise in the data, which can affect the quality of the inferences and results obtained by the networks. This interference may have critical implications in a wide range of applications, from medical misdiagnosis to safety decisions in autonomous vehicles, for example. Understanding and addressing this issue is critical to ensure the reliability of these technologies in real-world situations.

The specific problem to be addressed in this study concerns understanding the impact of certain factors on the performance of neural networks based on convolutional models. As mentioned above, when information is acquired from an electronic sensor to digitize an image, this image is inevitably affected to a greater or lesser extent by noise, such as Gaussian noise. This interference, combined with the actual lighting conditions in which the image was captured, can have a more significant impact than might be imagined on the performance of various neural network models. This can lead to the networks generating incorrect or insufficiently accurate results [6].

In addition to exploring the implications of noise on the performance of CNNs, this study aims to closely analyze the impact that different lighting conditions can have on their performance. In fact, illumination variability is a critical factor in many real-world applications, where images may be captured in environments with varying or insufficient illumination [7].

Through the evaluation of the networks under various brightness conditions, solid conclusions can be drawn about their ability to adapt and produce accurate results in challenging lighting situations. This information will be essential to understanding how these network models can be used effectively in real-world applications where lighting conditions can vary considerably. By considering both noise and lighting conditions in this study, we seek to provide a comprehensive view of the robustness and versatility of these CNNs in various circumstances.

Despite the growing presence of CNNs in our daily lives, there is a surprising paucity of studies that thoroughly analyze the impact of factors such as noise and others on their performance and efficiency. This underscores the pressing need to investigate and better understand how these systems respond under adverse conditions. Improving the robustness of neural networks in the presence of adverse conditions would not only be beneficial from an academic point of view, but could also have a significant impact in critical sectors such as healthcare and the automotive industry, where accuracy and reliability are imperative [8].

The main contribution of this work is to analyze the impact of noise and changes in the brightness of images on the object detection performance of deep convolutional neural networks. This analysis is based on an accurate physical model of noise and brightness for imaging CMOS sensors. Qualitative and quantitative assessments of the relative object detection performance of the deep networks are carried out, along with a discussion of the results.

The remainder of this paper is organized as follows. Related works are considered in Section 2. After that, Section 3 describes the physical model of noise and brightness for imaging CMOS sensors. The experiments that have been carried out are presented in Section 4. Finally, the conclusions of this work are detailed in Section 5.

## 2. Related Works

The images can be hindered by several factors, particularly by the influence of varied types of sensor noise. One of the possible reasons can be found in the constraints that image obtainment devices manifest [9,10,11]. Consequently, the input pixel values may be altered, and this behaviour may have an impact on the performance achieved by those methods that accomplish different tasks, such as background segmentation or classification. In order to analyze the impact of these noises on the performance of methods of different kinds, several studies have been carried out.

For example, the performance of different foreground object detection methods was studied when the input images were affected under diverse quantities of Gaussian and uniform noise [12]. Moreover, a certain number of situations were stated where the addition of noise to the input image might be beneficial in order to alleviate the constraints of a method. The performance yielded by a total of nine methods from the state-of-the-art where the input images were corrupted with both noises were analyzed in that paper.

Several factors may hinder the result of the classification provided by a CNN. In particular, one of these drawbacks is the sensor noise. An analysis of the effects of noise in these kinds of neural networks was presented in [7]. The methodology detailed two noise models for current CMOS vision sensors that allow entering Poisson, Gaussian, salt-and-pepper, speckle and uniform noise as an origin of imperfections in the image acquisition devices. This way, synthetic noise can be added to an image by using the proposed methodological framework to imitate usual sources of image distortion. With that suggested framework, each kind of noise type was combined with a bright scale factor to simulate images with low lighting conditions, and their impact on the classification performance of several state-of-the-art CNNs pretrained models was studied. The results showed that Gaussian and uniform noise have a moderate effect; speckle and salt-and-pepper noise, together with the level of brightness, could significantly decrease the classification performance; while Poisson noise did not have a substantial impact on the performance.

Another of the possible noises is the linear motion blur. Its impact in the performance of CNNs was evaluated by proposing a realistic vision sensor model to generate a linear motion blur effect in raw input images. By using this methodology, the classification performance of different pretrained CNNs was studied and the obtained results demonstrated that the more the displacement the more the degradation of the performance as expected. However, although the angle of displacement does not have as much impact as the length, the performance is slightly deteriorated. It is interesting to observe how higher values of motion length produce a higher drop in CNNs performances and make it more sensitive to the motion angle. Moreover, angles close to odd multiples of 45º imply a more relevant drop of the performance. Regarding opposite angles, they achieve the same performance; however, conjugate angles do not provide the same performance [13].

## 3. Methodology

This section outlines the methodology developed for this study. It involves the creation of an accurate model for an imaging CMOS sensor (Section 3.1), the process for introducing synthetic noise into digital images based on this model (Section 3.2), and the description of how the performance of an object detection deep neural network degrades as the noise level increases (Section 3.3).

### 3.1. Sensor Noise Model

**Figure 1:**
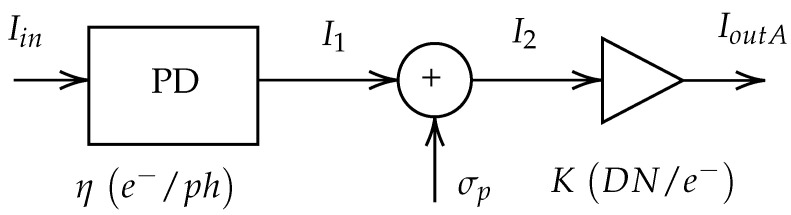
Model A. A conceptual model of a CMOS vision sensor, comprising a photodiode (PD) followed by a Poisson noise source (illustrated as a circular element). Subsequently, a conversion gain is represented as a triangular element. This model exclusively accounts for Poisson noise.

**Figure 2:**
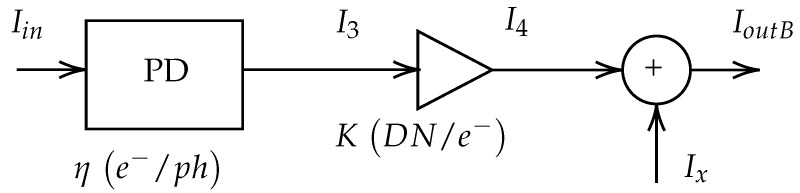
Model B. A conceptual model of a CMOS vision sensor, where a photodiode (PD) is succeeded by a conversion gain, represented as a triangular element, and followed by the introduction of a specific type of noise $I_{x}$ (depicted as a circular element).

We have defined two realistic noise models for a CMOS vision sensor, drawing inspiration from the European Machine Vision Association (EMVA) Standard 1288, which characterizes image sensors and cameras [14]. These models serve as the foundation for subsequent experiments to assess the performance of Convolutional Neural Networks (CNNs) under different noise sources. Figure 1 and Figure 2 represent these models, known as Model A and Model B, respectively:

- Model A simulates an imaging CMOS sensor with a single source of noise, namely Poisson type.
- Model B accounts for additional noise types, including Gaussian, speckle, salt-and-pepper, and uniform noises.

Both models are employed to maintain a realistic image creation process while isolating the sources of noise. The operation of the sensor, common to both models, is described based on Figure 1 and Figure 2. Images enter the device associated to a given amount of photons (ph) and interact with the photodiode (PD), which transforms the photons to electrons with a quantum efficiency factor ($\eta \;(e^{-}/ph)$). The result of this transformation carried out by the photodiode can be quantified as accumulated electrons. The Full Well Capacity (FWC) is defined as the maximum accumulation capacity for any given pixel. The equation that governs the transformation of photons into pixels reads:

(1) $$I_{1}=I_{3}=\eta I_{in}$$

where $I_{in}$ is in photons (ph), and $I_{1}$ is in electrons ($e^{-}$).

Shot noise, associated with photon counting errors, is assumed to distribute according to the Poisson distribution, and it is only considered by Model B. The signal corrupted by Poisson type noise (measured in electrons) features a mean parameter of the Poisson distribution equal to the noiseless signal $I_{1}$:

(2) $$I_{2}∼\mathrm{Poisson}\left(\lambda =I_{1}\right)$$

The subsequent steps include conversion gain, presented in both Model A and Model B, where accumulated electrons are converted to voltage (in microvolts, μV). In this step, the gain factor is noted χ ($\mu V/e^{-}$). The conversion from analog information to digital data converts the voltage signal into digital numbers (DN). This time the gain factor is noted ξ (DN/μV). These two gains are merged into an overall gain factor (K=χξ). Therefore, the computation of the theoretical result of the conversion from analog information to digital data reads as follows:

(3) $$I_{4}=KI_{3}$$

(4) $$I_{outA}=KI_{2}$$

However, the two conversions from electrons to a voltage signal, and then from analog information to digital data, are subject to additive noise, which is only considered in Model B. A signal level ($I_{x}$) is added before the output. If $I_{x}$ is measured in digital numbers (DN), then Model B indicates that the observed image can be expressed (in digital numbers DN) as follows:

(5) $$I_{outB}=I_{4}+I_{x}$$

### 3.2. Synthetic Noise Emulation

This subsection outlines a procedure for obtaining a noisy digital image based on the accurate models of noise discussed in Section 3.1. The noiseless pixel value in a digital image will be noted φ, measured in digital numbers (DN).

The simulation of Poisson noise is carried out by application of Model A. This involves the modification of the original noiseless signal by Poisson noise, where the signal is expressed in electrons (6). To achieve this, a division of the pixel value expressed in digital numbers by the overall gain *K* must be carried out, in order to yield the pixel value expressed in electrons. Moreover, low illumination situations may be modeled by applying a brightness scale factor *b*. Afterwards, a subsequent conversion to digital numbers is performed. The noisy pixel value obtained by the imaging device (${\hat{\varphi }}_{Poisson}$) can be calculated considering the original pixel value φ, as indicated next:

(6) $${\hat{\varphi }}_{p}=K\;\mathrm{Poisson}\left(\frac{b\varphi }{K}\right)$$

Model B is utilized to generate synthetic noise. Various types of synthetic noise representing common degradation mechanisms in digital images, including Gaussian (g), salt-and-pepper (sp), and uniform (u) noise, are considered. The resulting noisy pixel value $\tilde{\varphi }$ is determined based on the type of synthetic noise introduced.

Gaussian noise is expressed as:

(7) $${\tilde{\varphi }}_{g}=b\varphi +\mathrm{Gauss}\left(0,\sigma_{g}^{\prime }\right)$$

where *b* stands for the brightness scale, while $\sigma_{g}^{\prime }$ denotes the standard deviation. Common sources of Gaussian noise include intrinsic circuit noise and an elevated operating temperature.

A probability mass function is employed to model salt-and-pepper noise:

(8) $$P\left({\tilde{\varphi }}_{sp}\right)=\left\{\begin{array}{cc} p_{0} & \mathrm{if}\;{\tilde{\varphi }}_{sp}=0\\ p_{1} & \mathrm{if}\;{\tilde{\varphi }}_{sp}=255\\ 1-p_{0}-p_{1} & \mathrm{if}\;{\tilde{\varphi }}_{sp}=b\varphi \end{array}\right.$$

with probabilities $p_{0}$ for black pixels and $p_{1}$ for saturated pixels, respectively. The function can be simplified by assuming that $p_{0}$ and $p_{1}$ are equal, i.e., $p_{0}=p_{1}$.

Uniform noise is defined as:

(9) $${\tilde{\varphi }}_{u}=b\varphi +\mathrm{Uniform}\left(-\Delta ,\Delta \right)$$

where Δ specifies the extremes of the valid values for the corrupted image, drawn uniformly at random from the interval [−Δ,+Δ].

These Equations (6)–(9) must be employed for each channel (red, green, and blue) of the original image in order to obtain its corrupted version.

### 3.3. Object Detection Performance Degradation

Here, we describe how the performance of an object detection deep neural network degrades as the amount of noise present in the input image increases. Let us note ζ the noise level. This corresponds to a different parameter depending on the noise type, $\zeta \in \left\{K,\sigma_{g}^{\prime },p_{0},\Delta \right\}$ for Poisson, Gaussian, salt-and-pepper, and uniform noise, respectively. Also, let us remember that the brightness scale factor is noted *b*.

Then the performance of an object detection neural network can be expressed as a function of the noise level ζ and the brightness scale factor *b*, $A\left(\zeta ,b\right)$. Maximum performance should be attained for zero noise:

(10) $$A\left(0,b\right)\geq A\left(\zeta ,b\right),\;\forall \zeta \geq 0$$

Moreover, the performance should decrease as the noise level increases:

(11) $$\zeta_{0}\leq \zeta_{1}\Rightarrow A\left(\zeta_{0},b\right)\geq A\left(\zeta_{1},b\right)$$

The specific characteristics of the performance function A must be determined by experimentation for various object detection networks.

## 4. Experimental Results

**Figure 3:**
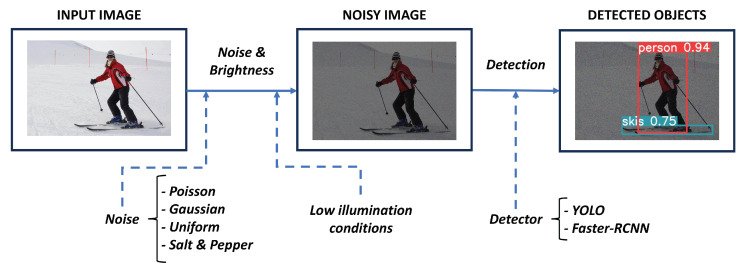
Schema of the proposed methodology. A raw image is contaminated with a source of noise and low illumination conditions are applied. The obtained noisy image from that process is supplied to a detector method in order to locate and classify the objects presented in that image.

Figure 3 offers a graphical abstract of how the study proposed in this work was conducted. First, the raw image is contaminated with a source of noise. Additionally, a brightness scale factor is applied to emulate low-illumination conditions. Therefore, after both noise and brightness processes, a noisy image is obtained. Next, that image is supplied to a detector method in order to locate and classify the objects presented in that image. Once the detections of all tuned configurations have been performed, a fair comparison has been carried out by using different well-known metrics. Finally, the obtained results from that comparison can be discussed.

This study aims to analyze the effect of the most relevant sources of sensor noises. With this intent, a set of experiments was carried out, and the obtained results are shown in this section. First of all, the considered methods are described in Section 4.1. Next, the selected dataset is detailed in Section 4.2. Then, the parameter configuration is presented in Section 4.3. At last, results are depicted in Section 4.4 and Section 4.5.

### 4.1. Methods

YOLO (You Only Look Once) and Faster R-CNN (Faster Region-based Convolutional Neural Network) are the neural network models that we will use for the battery of experiments. These architectures excel in their ability to identify and locate objects in a variety of scenarios, even in situations with brightness variations and Gaussian noise.

However, it is critical to understand that YOLO and Faster R-CNN, while sharing the purpose of object detection, differ in their approach and operation [15]. While YOLO excels in speed and efficiency in addressing real-time detection, Faster R-CNN offers a higher level of accuracy at the cost of greater computational complexity [16]. This distinction in performance and efficiency will be a key element in our evaluation, as it will determine which of the two architectures is more suitable for object detection in images with variations in brightness and Gaussian noise.

For the different experiments, different versions of these models have been used. As for YOLO, the pre-trained versions of YOLOv5nu, YOLOv5mu, YOLOv5xu [17], as well as other more current versions such as YOLOv8n, YOLOv8m, and YOLOv8x [18] have been used. Among these versioned versions of the YOLO model, YOLOv8 outperforms YOLOv5 in accuracy, achieving 54.2% average accuracy on the COCO dataset compared to 50.5% for YOLOv5. Both are suitable for real-time applications, with YOLOv5 offering higher FPS on the CPU, but YOLOv8 being preferable on some GPUs. The ‘n’ version of YOLOv8 is optimal for embedded devices such as Jetson Nano. In summary, YOLOv8 is more accurate, while YOLOv5 is faster on the CPU and YOLOv8 is preferable on some GPUs and embedded devices. Both mentioned YOLO versions v5 (https://github.com/ultralytics/yolov5, accessed on 11 December 2023) and v8 (https://github.com/ultralytics/ultralytics, accessed on 11 December 2023) are extracted from the Ultralytics library (https://github.com/ultralytics, accessed on 11 December 2023).

For the Faster R-CNN [19] models we have used the Pytorch-Torchvision library (https://pytorch.org/vision/main/models/faster_rcnn.html, accessed on 11 December 2023), making use of the Faster R-CNN ResNet50 FPN V2 (https://pytorch.org/vision/main/models/generated/torchvision.models.detection.fasterrcnn_resnet50_fpn_v2.html, accessed on 11 December 2023) and Faster R-CNN Mobilenet V3 Large FPN (https://pytorch.org/vision/main/models/generated/torchvision.models.detection.fasterrcnn_mobilenet_v3_large_fpn.html, accessed on 11 December 2023) versions, both with pre-trained weights. As for the differences between these versions, Faster R-CNN ResNet50 FPN V2 stands out for its high accuracy in object detection, thanks to the complex ResNet50 FPN architecture. However, it requires more computational resources, which may affect its speed. On the other hand, Faster R-CNN MobileNet V3 Large FPN focuses on speed and is ideal for real-time applications on resource-constrained devices, although its accuracy may be slightly lower due to its lighter architecture. In summary, ResNet50 offers accuracy, while MobileNet V3 is fast and efficient on resource-constrained devices.

### 4.2. Dataset

The COCO dataset, with its diversity of natural scenarios, detailed labels, and divisions for detection tasks, represents an essential resource in our computer vision research. COCO contains an extensive collection of about 300,000 images, meticulously selected to represent diverse and realistic natural settings. Of the more than 200,000 images available, more than 80 different object categories have been thoroughly labeled. This accurate labeling allows for detailed analysis of a wide range of objects, improving the robustness of our experiment. This provides a rich and varied dataset for our research. COCO dataset offers different versions of the dataset [20] and it has been divided into essential subsets for detection tasks. These subsets include training, validation, and test sets, each accompanied by corresponding annotations.

In this work, we have used the 2017 Val images dataset (http://images.cocodataset.org/zips/val2017.zip, accessed on 11 December 2023), which is composed of 5000 images with a size of approximately 1 GB, and their annotations (http://images.cocodataset.org/annotations/annotations_trainval2017.zip, accessed on 11 December 2023).

### 4.3. Parameter Selection

In order to establish a fair comparison between YOLO and Faster R-CNN models, their parameters were fixed to the same values. This way, the parameter object confidence threshold for detection conf for YOLO models and $box_{s}core_{t}hresh$ for Faster R-CNN models were fixed to 0.5.

Regarding the images, each color channel of each pixel will have a value within the interval [0, 255], so 8-bit encoded images are assumed.

Respecting the source of noise and brightness, the number of possible tuned configurations is enormous, as can be deduced from Section 3. This way, a parameter analysis has been established to obtain realistic results and a set of enough experiments that allow us to deduce solid conclusions. The tuned parameters and their description are as follow:

- *b*: The brightness scale factor emulates illumination conditions by controlling the minimum and maximum values of the image. The tuned values for this parameter *b* have been selected from the interval 0.1 to 1.0 with a step of 0.1. With this configuration, the lower the value of *b*, the darker the noisy image.
- *K*: The image sensor gain that converts electrons into digit values is represented by this parameter, which is exclusively dependent on the sensor performance. With the aim of analysing realistic scenarios, different commercial vision sensor data-sheets have been collected [21,22,23,24], where *K* goes from 0.01 to 0.1 $DN/e^{-}$. Furthermore, in order to study those more complex situations, higher values for *K* have been considered.
- $\sigma_{g}^{\prime }$: The standard deviation modulates the quantity of Gaussian noise. The higher the value of $\sigma_{g}^{\prime }$, the noisier the image. While the read-out noise of most commercial vision sensors is less than 1 DN when 8-bit encoding is used, values from 0 to 22.5 DN with a step of 2.5 DN have been considered.
- Δ: The limit range value establishes the minimum and maximum values that define the uniform noise that can be reached. The parameter Δ manages both values by considering the range [−Δ,Δ] to introduce additive noise. The chosen values for this parameter are in the interval from 0 to 22.5 DN with a step of 2.5 DN.
- *p*: This parameter represents the probability of having a pixel affected by salt-and-pepper noise. It has been considered that the likelihood for salt is precisely the same for pepper, so that, $p_{0}=p_{1}=p$ (see Section 3.2). The selected values for this parameter go from 0.00 to 0.27 with a step of 0.03.

Table 1 summarizes the parameter values which form the set of tuned configurations.

### 4.4. Qualitative Results

**Figure 4:**
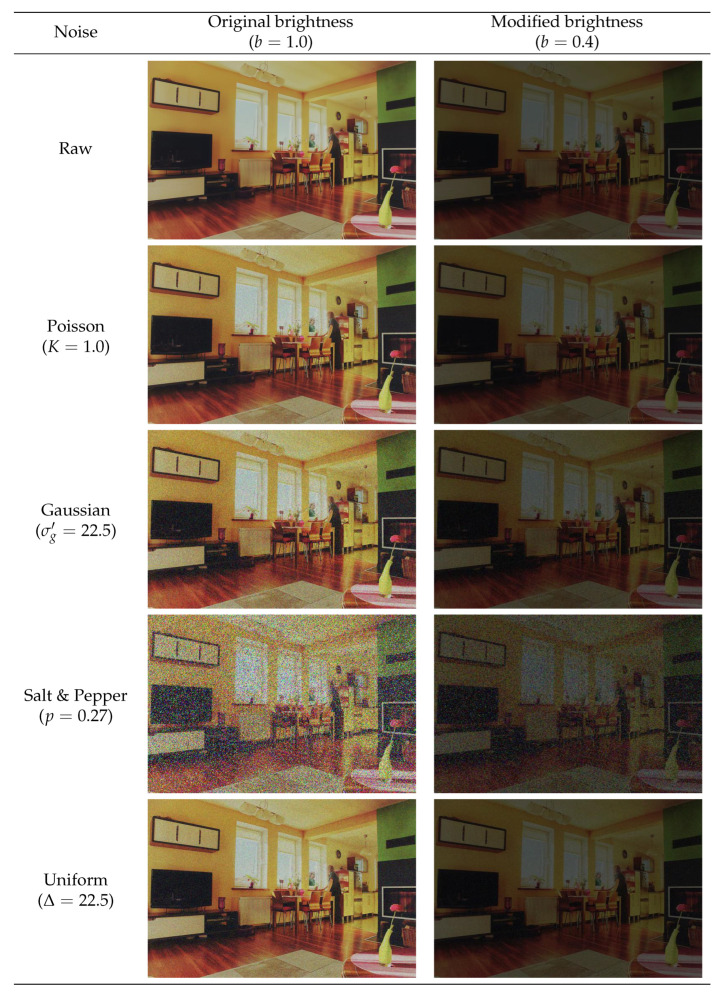
Image 139 without and with several quantities of different noises and bright scale configurations.

The different types of noise considered in this work have their nature. In order to provide a better comprehension of their incidence and low illumination conditions on the images, Figure 4 details an example of the effect they produce on an input raw image.

**Figure 5:**
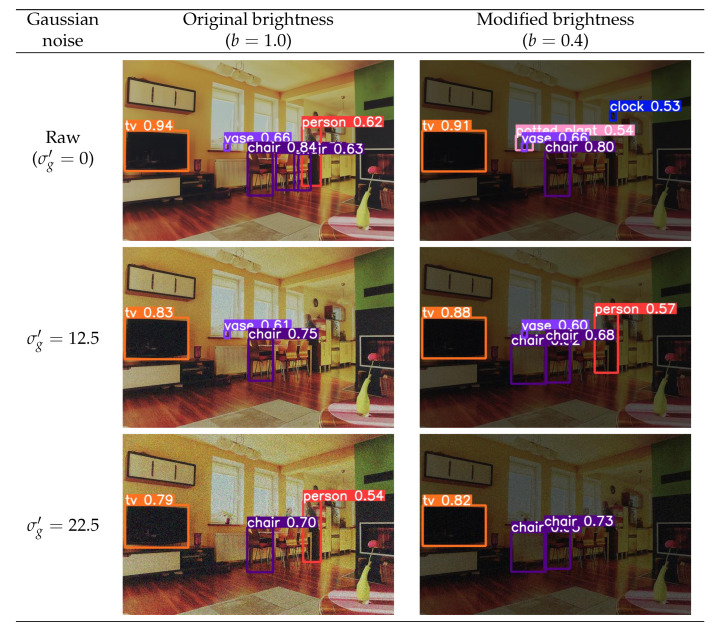
Image 139 without and with several quantities of **Gaussian noise** and bright scale configurations. Bounding boxes represent the object detections that the YOLOv5nu method has predicted. Each bounding box shows the class of the object and the confidence that the method has performed in that detection.

The performances yielded by the considered methods are compared from a qualitative point of view in this subsection. It aims to better comprehend the performance deterioration of the selected approaches. Without loss of generality, YOLOv5nu detector and Gaussian noise have been chosen for this purpose. The image selected is shown in Figure 5. It exhibits a room with plenty of objects that the methods may detect. The objects presented in the image are varied, such as chairs, tables, televisions, vases, potted plants, or people. As can be demonstrated, a noisier image does not have to provide a worse detection. In fact, a low quantity of noise can even be beneficial to enhance the detection. This remark can be observed with the clock: it is not detected in the raw image, but it is well detected when low-illumination conditions are applied.

However, in general, a more noisy image produces fewer detections and lower confidence in the model. As shown, the television is well detected in all images; nevertheless, the higher the quantity of noise, the lower the confidence of the method in that detection. This effect is more visible in the case of the vase, which is recognized in the absence of noise or with a low quantity of noise, but it is not detected when the noise is much higher.

This same behavior of the detections occurs when low illumination conditions are presented in the image. As can be observed, the person is better detected when brightness has not been modified.

These observations are presented similarly for the rest of the different considered models. Depending on the intrinsic characteristics of the selected model, the noise and the brightness of the input image, the model can detect a specific object in that image well or not.

### 4.5. Quantitative Results

In order to measure the performance of each method and establish a fair comparison between them, several well-known metrics have been considered for that purpose. In the context of the detection of objects presented in images, the utilization of Average Precision (AP) metric is useful to evaluate the effectiveness of the predictions.

Before going into the details of the evaluation, it must be highlighted what AP entails. In the field of object detection. AP is a performance metric used to measure the ability of a model to detect and locate objects in an image [25] and it considers two crucial aspects:

- Detection accuracy: This component evaluates how many of the detected objects are actually relevant. It is essential to determine the model’s ability to identify objects of interest under varying conditions, such as noise and brightness.
- Location accuracy: Accuracy in the location and size of detected objects is another essential element of the AP calculation. This is crucial to evaluate the ability of the model to not only detect objects but also to accurately localize them.

The AP metric provides a complete and detailed view of the model’s performance in terms of object detection and localization, which is essential to understanding how it performs against modifications introduced in the experiments.

Within the field of object detection, in addition to the AP metric, the mean Average Precision (mAP) metric is frequently used, which is a well-known metric that provides a more comprehensive evaluation by averaging the AP values obtained on different classes or categories of objects. The behaviour of mAP can be defined as:

- Calculation of AP by class: First, the AP value is calculated for each class of object being detected. This involves measuring the detection and localization accuracy specifically for that category.
- Average AP: Once the AP has been calculated for each class, these values are averaged to obtain the mAP. This average takes into account the detection and localization efficiency across all object categories, providing an overall assessment of the model.

The results obtained in the different experiments of this work are based on these metrics AP and mAP mentioned.

The AP measures the accuracy of the model by calculating the average accuracy value for the recovery value from 0 to 1, based on the Intersection over Union (IoU), which is a measure that evaluates the overlap between the predicted area and the true annotation area, i.e., how much the boundary predicted by our model overlaps with the boundary of the real object in the image.

Another concept that we should comment on, and to which we have made reference, is the accuracy itself. The accuracy simply measures how accurate the predictions made by our model are based on the IoU obtained from our detection, i.e., the percentage of predictions on object detections that are correct. In this work, the percentage of detections whose IoUs are 50% (0.5) or higher have been considered. This IoU threshold can be varied according to how strict we want the evaluation of the detections of our model to be, since all the detections that have an IoU lower than the established threshold will be discarded as possible true predictions.

Our evaluation code disaggregates between true positive (TP), true negative (TN), false positive (FP), and false negative (FN) cases. Then, for each object category, we calculate the precision at different IoU thresholds as mentioned above and average it based on the number of thresholds, obtaining the AP.

(12) $$Precision=\frac{TP}{TP+FP}$$

The advantage of using mAP lies in its ability to evaluate an object detection model more comprehensively, taking into account performance on multiple classes of objects rather than considering only one. This is especially relevant in practical applications where it is common to detect and locate various objects in an image. The use of mAP provides a global assessment of how the model performs in both conditions (detection accuracy and localization accuracy) across multiple object categories. This further enriches the understanding of the effectiveness of the model. Additionally, ground-truth objects are categorized into *small*, *medium*, and *large* according to their area measured as the number of pixels in the segmentation mask. In this way, a better understanding of the performance detection may be reported. More details about detection evaluation can be found in the COCO dataset website (https://cocodataset.org/#detection-eval, accessed on 11 December 2023).

**Figure 6:**
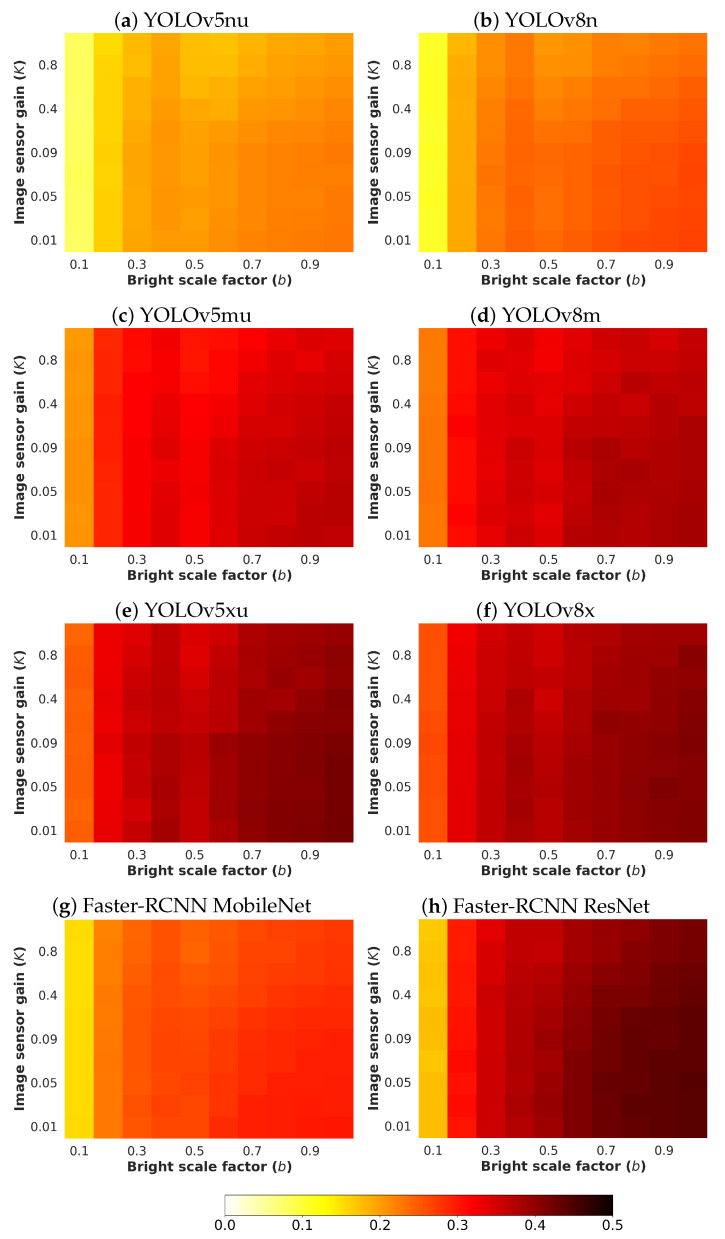
Heatmap of mAP for considered detectors where images have been degraded introducing different levels of bright scale and **Poisson noise**.

**Figure 7:**
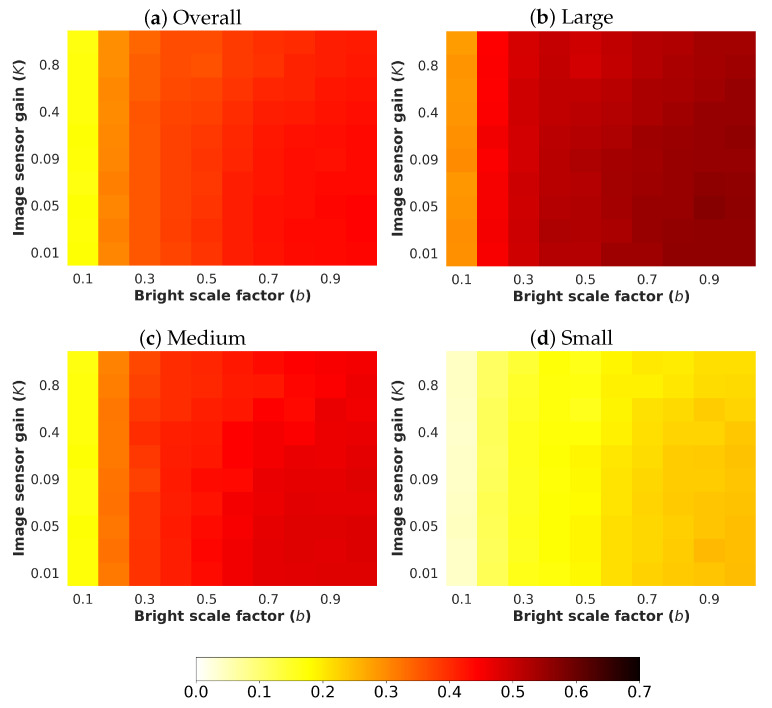
Heatmap of mAP for **Faster-RCNN ResNet** according to the size of the ground-truth objects where images have been degraded introducing different levels of bright scale and **Poisson noise**.

**Figure 8:**
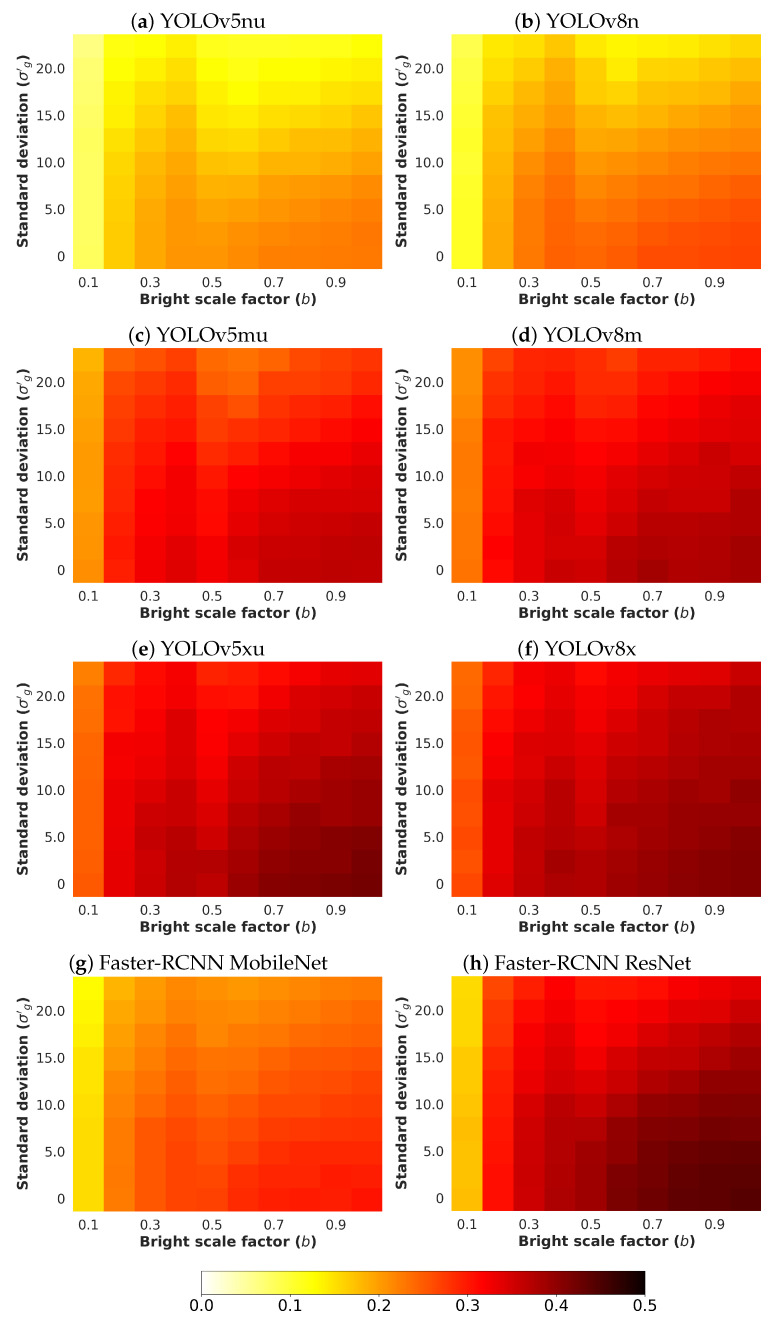
Heatmap of mAP for considered detectors where images have been degraded introducing different levels of bright scale and **Gaussian noise**.

**Figure 9:**
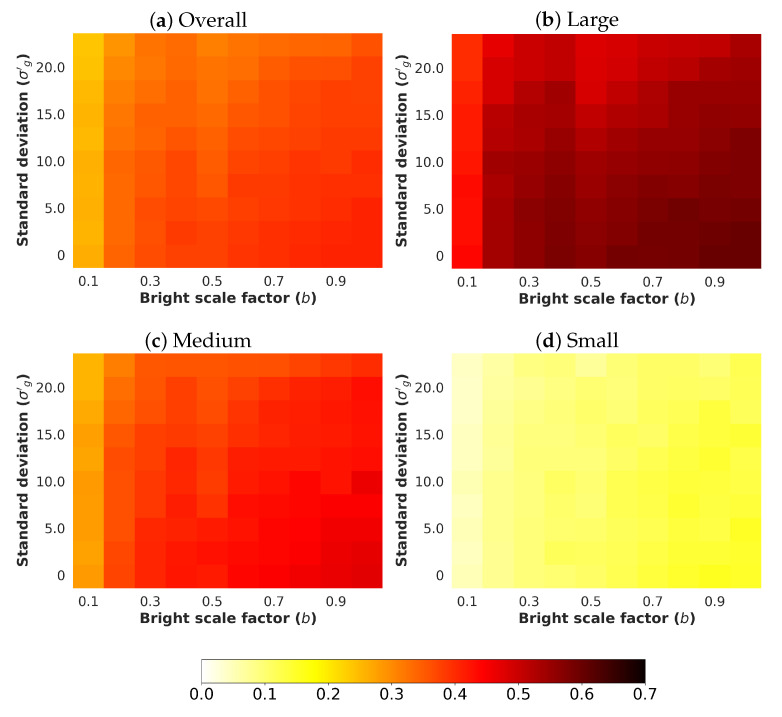
Heatmap of mAP for **YOLOv8x** according to the size of the ground-truth objects where images have been degraded introducing different levels of bright scale and **Gaussian noise**.

**Figure 10:**
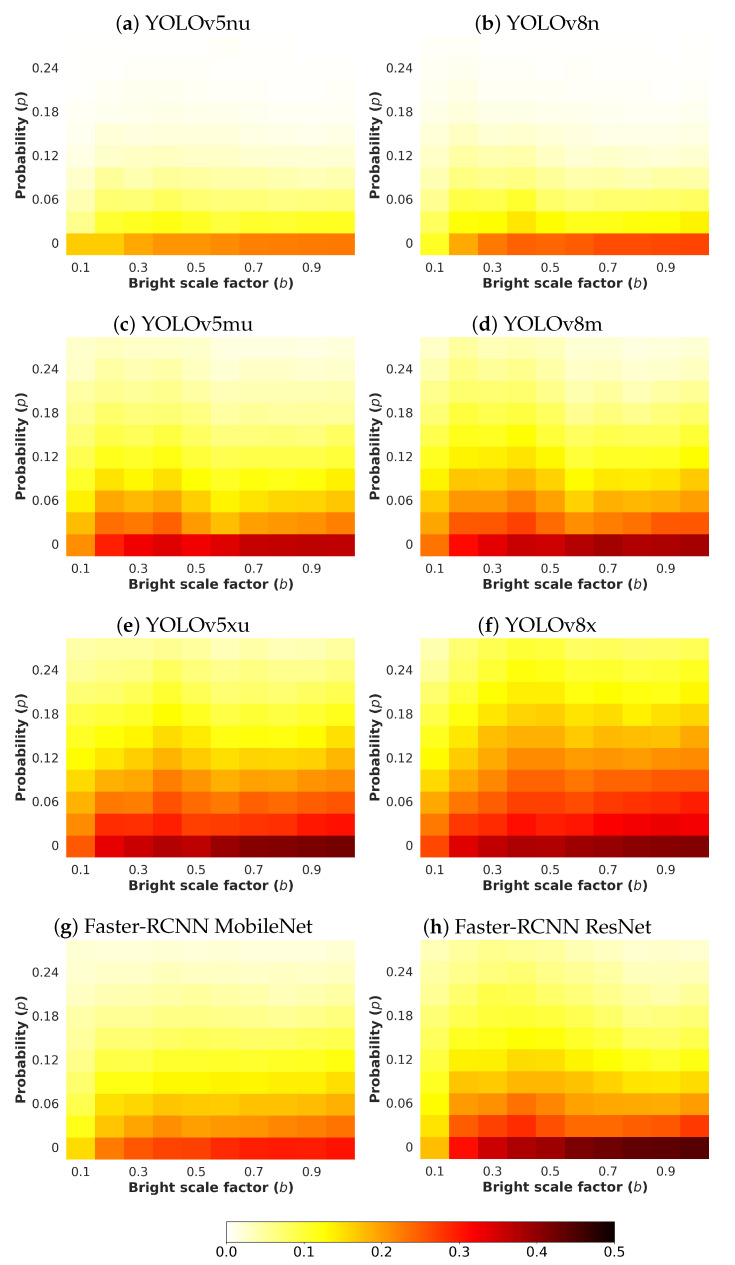
Heatmap of mAP for considered detectors where images have been degraded introducing different levels of bright scale and **salt-and-pepper noise**.

**Figure 11:**
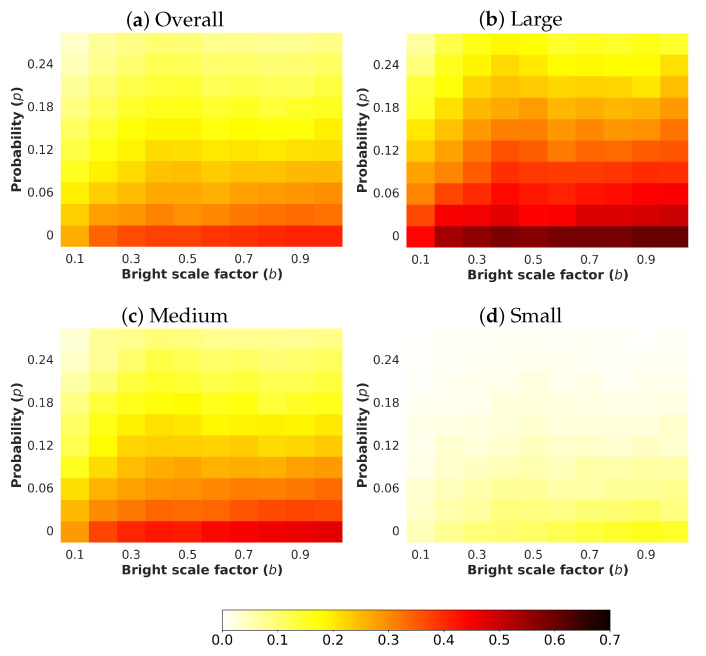
Heatmap of mAP for **YOLOv8x** according to the size of the ground-truth objects where images have been degraded introducing different levels of bright scale and **salt-and-pepper noise**.

**Figure 12:**
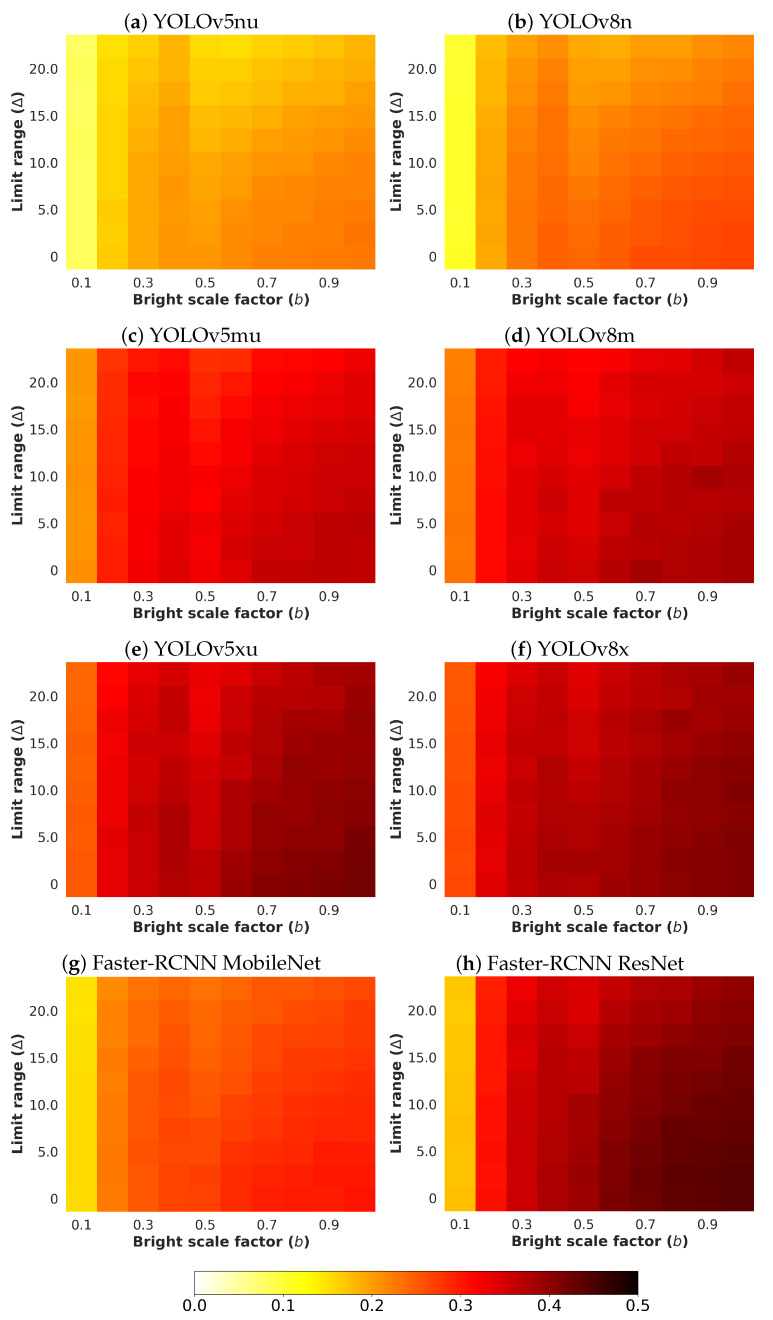
Heatmap of mAP for considered detectors where images have been degraded introducing different levels of bright scale and **uniform noise**.

**Figure 13:**
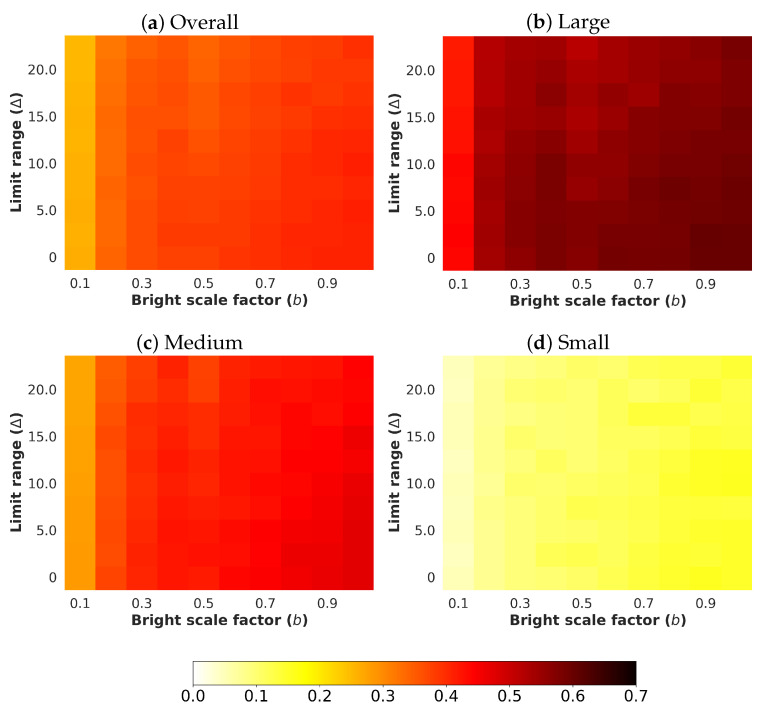
Heatmap of mAP for **YOLOv8x** according to the size of the ground-truth objects where images have been degraded introducing different levels of bright scale and **uniform noise**.

Next, an analysis and comparison have been performed for each type of considered noises. For each type of noise, the performance of each detector (methods are detailed in Section 4.1) is shown according to the quantity of noise and the brightness of the image (considered values are described in Section 4.3). The performance is reported in terms of mAP, which is considered the primary challenge metric by the COCO dataset. This measure provides values between 0 and 1, where higher is better. To better understand the effect of noise and brightness, the performance yielded by each method for each kind of noise is reported using a heatmap, where the performance for each configuration of noise and brightness is detailed. Two figures each from Figure 6, Figure 7, Figure 8, Figure 9, Figure 10, Figure 11, Figure 12 and Figure 13 show the results for Poisson, Gaussian, salt-and-pepper, and uniform noises, respectively. For each type of noise, the first figure describes the performance of the methods by considering the overall size of the ground-truth objects, while the second one details the performance of a selected method across small, medium, and large sizes. With the aim of not overcharging this study with the performance across scales small, medium, and large sizes for all selected methods, only the most significant method for each noise is exhibited. Note that the configuration with b=1.0 corresponds with no synthetic low-illumination conditions.

It must be highlighted that the first row, from bottom to top of each heatmap, shows the performance of that method when no noise is introduced, except for Poisson noise. This way, this row will be the same for each analyzed noise. Moreover, note that the configuration with b=1.0 (no synthetic low illumination conditions) and no noise matches the configuration where the raw original images are supplied to the detectors. In the case of Poisson noise, there is no raw without noise because this noise is multiplicative, not additive like the remaining noises.

In general terms, the results from Figure 6, Figure 7, Figure 8, Figure 9, Figure 10, Figure 11, Figure 12 and Figure 13 demonstrate, as expected, the lower the illumination conditions (bright scale factor), the lower the performance. Also predictable is that the performance deterioration is proportional to the addition of noise for every bright scale value. Regarding the performance according to the size of the ground-truth objects, the size influences the efficiency of the methods: the larger the size of the objects, the better they are detected and well-classified. The difficulty detecting small objects must be highlighted, where the detector methods do not yield properly even in the absence of noise and adequate illumination conditions.

#### 4.5.1. Poisson Noise

The overall results for Poisson noise are shown in Figure 6. From this figure, it can be deduced that the Faster-RCNN ResNet model achieves the best performance. Furthermore, this degradation is more perceptible for the lowest values of the bright scale. In these cases, YOLOv5xu performs better.

The performance of the Faster-RCNN ResNet model, taking into account the categorization of the objects by their size, can be observed in Figure 7.

#### 4.5.2. Gaussian Noise

Figure 8 shows the results for Gaussian noise. As can be observed, YOLOv8x has the best performance. The performance degradation is proportional to the standard deviation $\sigma_{g}^{\prime }$ for every bright scale value. Moreover, this deterioration is more noticeable in the lowest-illumination conditions. This is because the quantity of noise introduced for each pixel is comparable with the maximum pixel value of the input image.

Regarding the performance according to the size of the ground-truth objects, the size influences the efficiency of the methods, as can be observed in Figure 9, where the performance of YOLOv8x is shown.

#### 4.5.3. Salt & Pepper Noise

Figure 10 exhibits the performances yielded by the methods for salt-and-pepper noise. It is interesting to observe the great impact that this noise has on the detections: from a certain value of *p*, depending on the method, the performance of the detectors drops drastically. The impact of this noise on the performance surpasses the effect of the brightness, which is practically non-existent.

YOLOv8x achieves the highest scores, and Figure 11 reports the results by size. As shown, the largest objects are well-detected for a considerable amount of salt-and-pepper noise, even under low illumination conditions; however, detecting the smallest objects involves serious difficulties.

#### 4.5.4. Uniform Noise

The results for uniform noise are shown in Figure 12. The results are similar to those obtained for Gaussian noise, although the impact of the uniform noise is lesser than the Gaussian noise. Again, YOLOv8x has the best performance.

Figure 13 exhibits the performance of YOLOv5xu according to the size of the ground-truth objects.

## 5. Conclusions

This paper presents a study of the impact of the most prevalent noise types and brightness alterations in images on the performance of object detectors. The exposed methodology proposes an accurate model of vision sensor noise in order to carry out the analysis of the noise types considered in this work: Poisson, Gaussian, salt-and-pepper, and uniform noise. The influence of the low illumination conditions accompanying each type of noise has also been studied. Several object detection methods have been selected such as different versions of YOLO v5 and v8, and Faster-RCNN.

Qualitative results demonstrate the need for a more comprehensive analysis due to the disparity of the predictions supplied by the detectors. This way, different configurations of noise and brightness have been tuned in conjunction with a set of 5000 images to form an exhaustive set of experiments. From a quantitative point of view, the experimental results conclude that, in general, the insertion of noise and/or a reduction of the brightness of the image has a negative incidence on the performance of the detector methods. However, there are situations where adding a small quantity of noise and/or reducing the illumination conditions may be beneficial to detect objects that are not detected in the raw input image.

This analysis might be helpful when designing systems composed of any object detector. In particular, the knowledge leveraged by this work could be most beneficial for systems dealing with environments featuring the presence of noise and/or low-illumination conditions.

## Figures and Tables

**Table 1:** Considered parameters and their possible values to study the performance of the different selected methods for diverse noises and illumination conditions.

Parameter		Value
Bright scale factor, *b*	=	{0.1,0.2,0.3,0.4,0.5,0.6,0.7,0.8,0.9,1.0}
Image sensor gain, Poisson noise, *K*	=	{0.01,0.03,0.05,0.07,0.09,0.2,0.4,0.6,0.8,1.0}
Standard deviation, Gaussian noise, $\sigma_{g}^{\prime }$	=	{0,2.5,5.0,7.5,10.0,12.5,15.0,17.5,20.0,22.5}
Probability salt and pepper noise, *p*	=	{0.00,0.03,0.06,0.09,0.12,0.15,0.18,0.21,0.24,0.27}
Limit range, uniform noise, Δ	=	{0,2.5,5.0,7.5,10.0,12.5,15.0,17.5,20.0,22.5}

## Data Availability

Publicly available datasets were analyzed in this study. This data can be found here: [20] (http://images.cocodataset.org/zips/val2017.zip, accessed on 11 December 2023), (http://images.cocodataset.org/annotations/annotations_trainval2017.zip, accessed on 11 December 2023). The source code developed for this work is also available (https://github.com/icai-uma/The-impact-of-noise-and-brightness-on-object-detection-methods, accessed on 11 December 2023).
